# Imaging Tumor Necrosis with Ferumoxytol

**DOI:** 10.1371/journal.pone.0142665

**Published:** 2015-11-16

**Authors:** Maryam Aghighi, Daniel Golovko, Celina Ansari, Neyssa M. Marina, Laura Pisani, Lonnie Kurlander, Christopher Klenk, Srabani Bhaumik, Michael Wendland, Heike E. Daldrup-Link

**Affiliations:** 1 Department of Radiology, Molecular Imaging Program at Stanford, Stanford University, Stanford, CA, United States of America; 2 School of Medicine, Tufts University, Medford, MA, United States of America; 3 GE Global Research Center, Research Circle, Niskayuna, NY, United States of America; 4 University of California, Berkeley, CA, United States of America; Case Western Reserve University, UNITED STATES

## Abstract

**Objective:**

Ultra-small superparamagnetic iron oxide nanoparticles (USPIO) are promising contrast agents for magnetic resonance imaging (MRI). USPIO mediated proton relaxation rate enhancement is strongly dependent on compartmentalization of the agent and can vary depending on their intracellular or extracellular location in the tumor microenvironment. We compared the T1- and T2-enhancement pattern of intracellular and extracellular USPIO in mouse models of cancer and pilot data from patients. A better understanding of these MR signal effects will enable non-invasive characterizations of the composition of the tumor microenvironment.

**Materials and Methods:**

Six 4T1 and six MMTV-PyMT mammary tumors were grown in mice and imaged with ferumoxytol-enhanced MRI. R1 relaxation rates were calculated for different tumor types and different tumor areas and compared with histology. The transendothelial leakage rate of ferumoxytol was obtained by our measured relaxivity of ferumoxytol and compared between different tumor types, using a t-test. Additionally, 3 patients with malignant sarcomas were imaged with ferumoxytol-enhanced MRI. T1- and T2-enhancement patterns were compared with histopathology in a descriptive manner as a proof of concept for clinical translation of our observations.

**Results:**

4T1 tumors showed central areas of high signal on T1 and low signal on T2 weighted MR images, which corresponded to extracellular nanoparticles in a necrotic core on histopathology. MMTV-PyMT tumors showed little change on T1 but decreased signal on T2 weighted images, which correlated to compartmentalized nanoparticles in tumor associated macrophages. Only 4T1 tumors demonstrated significantly increased R1 relaxation rates of the tumor core compared to the tumor periphery (p<0.001). Transendothelial USPIO leakage was significantly higher for 4T1 tumors (3.4±0.9x10^-3^ mL/min/100cm^3^) compared to MMTV-PyMT tumors (1.0±0.9x10^-3^ mL/min/100 cm^3^). Likewise, ferumoxytol imaging in patients showed similar findings with high T1 signal in areas of tumor necrosis and low signal in areas of intracellularly compartmentalized iron.

**Conclusion:**

Differential T1- and T2-enhancement patterns of USPIO in tumors enable conclusions about their intracellular and extracellular location. This information can be used to characterize the composition of the tumor microenvironment.

## Introduction

Ultra small superparamagnetic iron oxide nanoparticles (USPIO) have been developed as contrast agents for magnetic resonance (MR) imaging [1–5]. USPIO have several advantages over standard small molecular paramagnetic contrast agents. These include:

1. Higher relaxivity (magnetic moment) leading to higher sensitivity [6].
2. Specific tumor delivery via the enhanced permeability and retention (EPR) effect [7].
3. Possibility to chemically link diagnostic pharmacophores for targeted delivery [8].
4. Possibility to add other contrastophores for multi-modality imaging [9, 10].
5. Possibility to link therapeutic pharmacophores for simultaneous imaging and therapy [11–13].

Several USPIO compounds are currently translated to clinical applications [14–18]. It is important to understand their complex effect on tissue MR signal enhancement, which is highly dependent on the geometry and microscopic compartmentalization of the magnetic centers of the nanoparticles [19–21]. Intravenously injected USPIO initially distribute in the blood pool. Due to their large size, they are confined to the intravascular space in most organs. In tumors, USPIO slowly leak across the hyper-permeable endothelium of tumor micro vessels, accumulate in the tumor interstitium and are phagocytosed by tumor-associated macrophages [22, 23]. The transverse relaxivity (negative/dark signal effect on T2-weighted MR images) of the USPIO increases during interstitial and intracellular clustering processes, reaching a concentration-dependent maximum and followed by a plateau or a minor decline at very high tissue concentrations [24]. On the other hand, the longitudinal relaxivity (positive/bright signal effect on T1-weighted MR images) of USPIO is linearly related to the ability of the nanoparticles to interact with protons [25]. Previous studies noted high T1-signal of USPIO with intravascular compartmentalization and high T1-signal of USPIO in most solid tissues, including tumors [26], presumably due to decreasing proton-interactions throughout interstitial and intracellular-intralysosomal clustering processes.

Therefore, we hypothesized that regional differences in T1- and T2-relaxivities of USPIO *in vivo* may enable discrimination of different histopathological compartments within tumor tissue. While the T1- and T2-enhancement pattern of intracellular and extracellular USPIO has been studied extensively *in vitro* [20, 25], to our knowledge nobody has compared T1- and T2-enhancement pattern of intracellular and extracellular USPIO *in vivo*. Thus, the goal of our study was to compare the T1- and T2-enhancement pattern of intracellular and extracellular USPIO in mouse models of cancer and pilot data from patients. A better understanding of MR signal effects of intracellular and extracellular USPIO in tissues will facilitate non-invasive USPIO localization *in vivo*, enable conclusions about the composition of the tumor microenvironment and form the basis for related quantitative analyses.

## Materials and Methods

### Contrast Agents

Ferumoxytol (AMAG Pharmaceuticals, Cambridge, MA) is a colloidal solution of USPIO nanoparticles, which are composed of an iron oxide core and a semisynthetic carbohydrate coating of polyglucose sorbitol carboxymethyl ether with a hydrodynamic diameter of 30 nm [27]. Ferumoxytol is FDA-approved as an iron supplement for treatment of anemia in patients with chronic kidney disease [28] and has been used “off label” as an MR contrast agent by our group and others [1, 4, 14, 27]. The agent has an R1 relaxivity of 38 mM^-1^s^-1^, an R2 relaxivity of 83 mM^-1^s^-1^ (at 0.47 T and 39°C) and a plasma half-life of 14.5 hours in humans [1, 14, 29].

GEH121333 is a new core/shell USPIO agent with an iron oxide core and a hydroxyphosphonate-PEG shell. GEH121333 nanoparticles have an average hydrodynamic diameter of 22 nm, as measured by differential Light Scattering (DLS) with monodispersity, as measured by Field Flow Fractionation (FFF). GEH121333 nanoparticles have an R1 relaxivity of 17 s^-1^mM^-1^ and R2 relaxivity of 40 s^-1^mM^-1^ measured at 1.5 T and 40°C [30].

Gd-DTPA (Gadopentetate Dimeglumine, Magnevist, Bayer Shering Pharma AG, Berlin, Germany) is a standard, low molecular weight, gadolinium chelate with an R1 relaxivity of 3.8 mM^-1^s^-1^, an R2 relaxivity of 4.1 mM^-1^s^-1^ (at 0.47 T and 37°C) and a plasma half-life of 1.6 hours [31]. A dose of 0.2 mmol/kg was diluted in 100 μl normal saline and injected via an indwelling tail vein catheter.

### Comparison of iron oxide nanoparticle enhancement in tumors with and without central necrosis

Animal experiments were approved by Institutional Animal Care and Use Committee (IACUC) at the University of California San Francisco and Administrative Panel on Laboratory Animal Care (APLAC) at Stanford University and all animals were kept and treated in accordance to NIH and institutional guidelines. Two tumor models were used: To mimic a slow-growing malignancy, a transgenic MMTV-PymT model was used [32, 33]. MMTV-PymT mice develop spontaneous, multi-focal cancers of the mammary glands. Three MMTV-PymT mice were sacrificed at day 100, their tumors explanted, homogenized, pooled, and re-implanted into the mammary fat pads of 6 non-transgenic FVB/n mice. Explant transplantation was used to eliminate potential bias due to differences in tumor latency, tumor grade and number of tumors per animal. MMTV-PymT explants with sizes up to 1 cm do not typically show central areas of necrosis. To mimic a quickly growing, more aggressive malignancy with early central necrosis formation, 4T1 cells were implanted into mammary fat pads of 6 BALB/c mice. The tumor size was measured with a caliper every other day. Animals were referred to MR imaging once the tumors reached a diameter of 1 cm.

Six animals with 4T1 tumors and six animals with MMTV PyMT tumors underwent MR imaging under isoflurane anesthesia, using a 2 T Omega CSI-II animal scanner (Bruker Instruments, Fremont, CA) and a custom birdcage radiofrequency coil with a circulating deuterium oxide warming pad. MR images were obtained before, immediately after, 1 hour after and 24 hours post injection (p.i.) of ferumoxytol using following sequences: (i) T1 weighted spin echo (SE) 500/12 ms (TR/TE) sequences, (ii) T2* weighted gradient echo (GE) 240/10 ms (TR/TE) flip angle 30 degrees sequences and (iii) inversion recovery (IR) 3000/1500 ms (TR/TE) with increasing inversion times (70–3370 ms in increments of 300 ms). (i) and (ii) were acquired with a slice thickness of 2 mm, a field of view of 3x3 cm, and a matrix of 128x128, (iii) had same slice thickness and field of view but a matrix of 64x64. Ferumoxytol was injected at a dose of 0.5 mmol Fe/kg body weight (27.92 mg Fe/kg) in 100 μl normal saline via an indwelling tail vein catheter placed before animal imaging.

Inversion recovery fast GE images were used to calculate maps of relaxation rate for T1, R1 (1/T1), for each slice covering the tumor by fitting the image pixel values to the following equation: SI(TI) = 1–2 exp (-TI ˣ R1), where TI is inversion time. Regions of interest were drawn on the R1 maps to measure the entire tumor in all slices and the resultant data were averaged by weighting the individual slice values by the number of tumor pixels in that slice.

The trans-endothelial permeability of ferumoxytol across tumor micro vessels was calculated from 4T1 and MMTV-PymT tumors. We assume that the change in MR signal enhancement in tumor is a combination of signal change from contrast in the blood as well as in the interstitial space and that the blood concentration of ferumoxytol is roughly constant during the first hour after intravenous administration [22]. Dividing the R1 change in the tumor tissue by the R1 change in blood provided estimates of the relative tumor blood volume [34]. A blood concentration time curve was obtained by sampling blood from mice immediately (5 minutes), 60 minutes and 24 hours post injection of 0.5 mmol Fe/kg of ferumoxytol and by measuring R1 values by inversion spectroscopy. The trans-endothelial leakage rate of ferumoxytol in mmol (Fe) per unit time was obtained by dividing the tumor ΔR1 (R1 at 1 h minus R1 at 5 min) by our measured relaxivity of ferumoxytol.

### Evaluation of tumor enhancement with monodisperse iron oxide nanoparticles

To evaluate, whether the variable size of ferumoxytol nanoparticles was the reason for the observed differences in tumor enhancement, we performed an additional experiment in six mice MMTV-PyMT tumors. The tumors were allowed to grow up to a size of 1.5 cm, which lead to formation of a central necrosis as confirmed by fluid-equivalent central hyperintense tumor areas on T2-weighted MR images. MR images were obtained on a 7T animal MR scanner before iron oxide injection and at 24 and 48 h post injection (p.i.,) using the following pulse sequences: T1-weighted 3-dimensional (3D) fast spoiled gradient recalled acquisition (FSPGR) 6.6ms/1.4ms/2-30° (repetition time/echo time/flip angle) and T2*-weighted 3D FGRE 70ms/1.6–20.6ms/20°. All MR acquisitions were performed with a field of view of 4.5x4.5 cm and a slice thickness of 0.6 mm. MR data were analyzed by one independent investigator at Stanford using custom research software tool (Cinetool, GE Global Research Center). T1 and T2* relaxation times of tumors were calculated based on multi-flip-angle FSPGR and multi-echo FGRE images, converted to relaxation rates (R1 = 1/T1, R2* = 1/T2*). Statistical comparisons of ΔR1 relaxation rates of 4T1 and MMTV-PymT tumors was performed using a t-test with a p < 0.05 considered to be significant.

### Histology

After the last imaging sequence, animals were sacrificed and tumors were explanted and immersed in formalin. Sections were dehydrated and embedded in paraffin for cutting. Routine H&E staining was performed on sections to detect necrosis versus viable tumor tissue. Necrotic areas were identified by microscopic appearance of coagulative necrosis with sheets of homogenous clusters and dying and dead cells whereas viable tumor tissue was identified by microscopic appearance of intact cells. Histologic distribution of iron on Prussian blue stain was used to differentiate between compartmentalized and free iron. Blue staining in a compartmentalized fashion indicated intracellular iron whereas diffuse blue staining not in a cellular distribution indicated extracellular iron.

Formalin-fixed paraffin embedded (FFPE) whole tumor tissue sections of GEH121333 injected animals were deparaffinized with Histochoice clearing agent (Amresco, Solon, OH), rehydrated by a series of alcohol washes, and processed for iron stains and antigen retrieval. Immunohistochemistry (IHC) detection of tumor associated macrophages (TAM) and GEH121333 in tumor tissue was performed by an antigen retrieval method developed specifically for FFPE tissues. In brief, slides were incubated in citrate buffer for 20 minutes followed by 0.3% Triton X-100/EDTA for 20 minutes and blocked against non-specific binding by incubating with blocking solution (10% goat serum/PBS) for 1–2 hours at room temperature. A mixture of two primary antibodies containing (1) anti-PEG, rabbit-derived (Epitomics Inc., Burlingame, CA) and (2) rat anti-mouse F4/80 (Serotec, Oxford, UK) was applied on the sections at a dilution of 1:100 and left overnight at 4°C in PBS/10% goat serum. For indirect detection of bound primary antibodies, species-specific Cy3 or Cy5-conjugated secondary antibodies (Jackson ImmunoResearch Lab Inc., West Grove, PA) were used at a dilution of 1:200. The sections were further stained with 4',6-diamidino-2-phenylindole (DAPI) to identify cell nuclei.

### Clinical Translation

As a proof-of-concept for clinical translation of the observed findings, we investigated four patients with bone tumors with ferumoxytol-enhanced MRI as a part of a larger clinical trial aimed at improving tumor staging with iron oxide based contrast agents [35]. We selected tumors with macroscopic areas of T2-hyperintense central necrosis on pre-contrast MR images for this part of our study. The study was approved by the committee for human research and the cancer center at Stanford University and was conducted under an investigator initiated Investigational New Drug (IND) with the Food and Drug Administration (FDA) for off label use of ferumoxytol as an MR contrast agent. Following written informed consent, patients underwent MRI on a 3 T clinical MR scanner (Discovery MR750, GE Healthcare, Waukesha, WI, USA) using a 32 channel torso phased array coil. Pulse sequences comprised a fat-saturated T1 weighted 3D-spoiled gradient recalled echo (SPGR) sequence (TR/TE/flip angle = 12ms/3.8ms/15) and a T2* weighted enhanced fast gradient echo 3D (Efgre3D) sequence (TR/TE/flip angle = 70.4/2-30ms/25). Imaging was performed before, 15 minutes after and 24 hours p.i. of ferumoxytol at a dose of 5 mg Fe/kg (0.1 mmol Fe/kg). Other parameters included a slice thickness of 5 mm and a matrix size of 196x256 pixels. MRI imaging was correlated with other available imaging modalities to include CT, Gd-DTPA enhanced MRI and FDG-PET scans.

## Results

### Ferumoxytol enhancement in tumors with and without central necrosis

**Fig 1:**
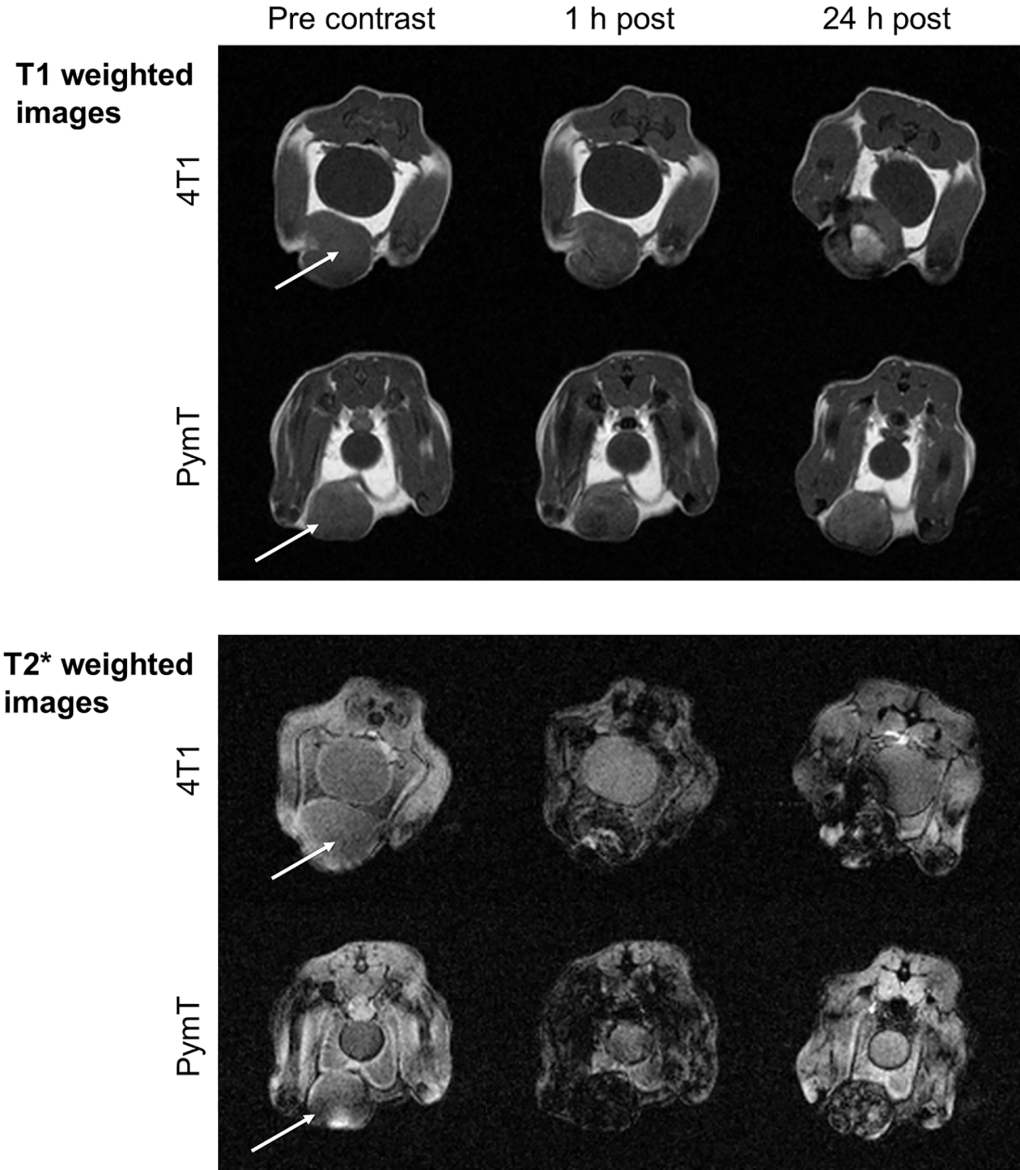
Ferumoxytol nanoparticles delineate early necrosis on MR images. Axial T1- and T2*-weighted MR images of 4T1 and PymT tumors in mice before, 1 hour and 24 hours post injection (p.i.) of ferumoxytol at a dose of 0.5 mmol Fe/kg. Note homogenous tumors on pre-contrast images with relatively uniform blood pool enhancement at 1 h p.i.. 4T1 tumors demonstrate marked central T1-enhancement at 24 h p.i..

**Fig 2:**
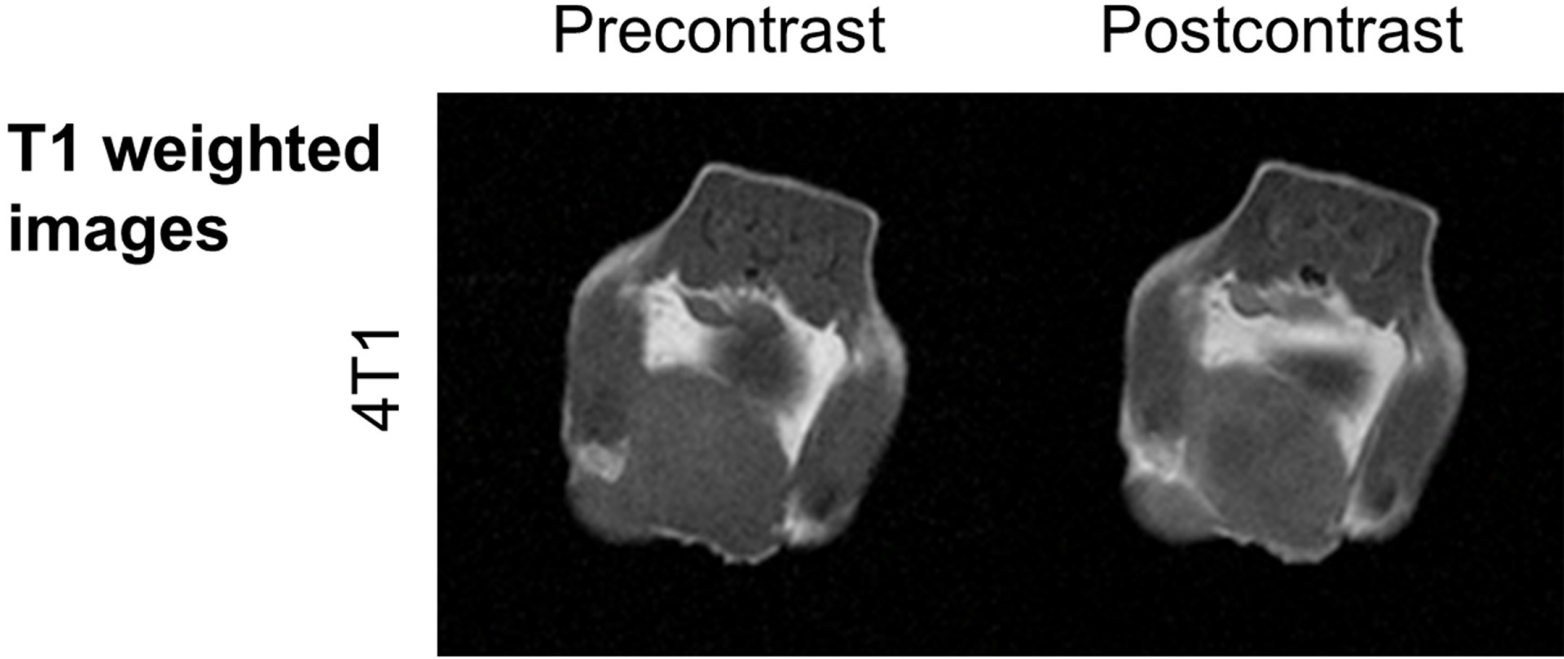
Gadopentetate does not delineate early necrosis. Axial T1 weighted MR images of the same 4T1 tumor as in Fig 1 at 60 seconds after intravenous injection of Gd-DTPA at a dose of 0.2 mmol/kg. Note homogenous tumor morphology with only slightly increased enhancement of the tumor periphery compared to the tumor center.

Both MMTV-PymT and 4T1 tumors appeared homogenous and featureless on pre contrast T1 and T2* weighted MR images (Figs 1 and 2). There was no evidence of intratumoral inhomogeneity or central T2* hyper intensity to suggest central tumor necrosis. Injection of Gd-DTPA into animals with 4T1 tumors yielded faint primarily peripheral tumor enhancement that in some animals, but not consistently, showed a central core with decreased perfusion (Fig 2).

**Fig 3:**
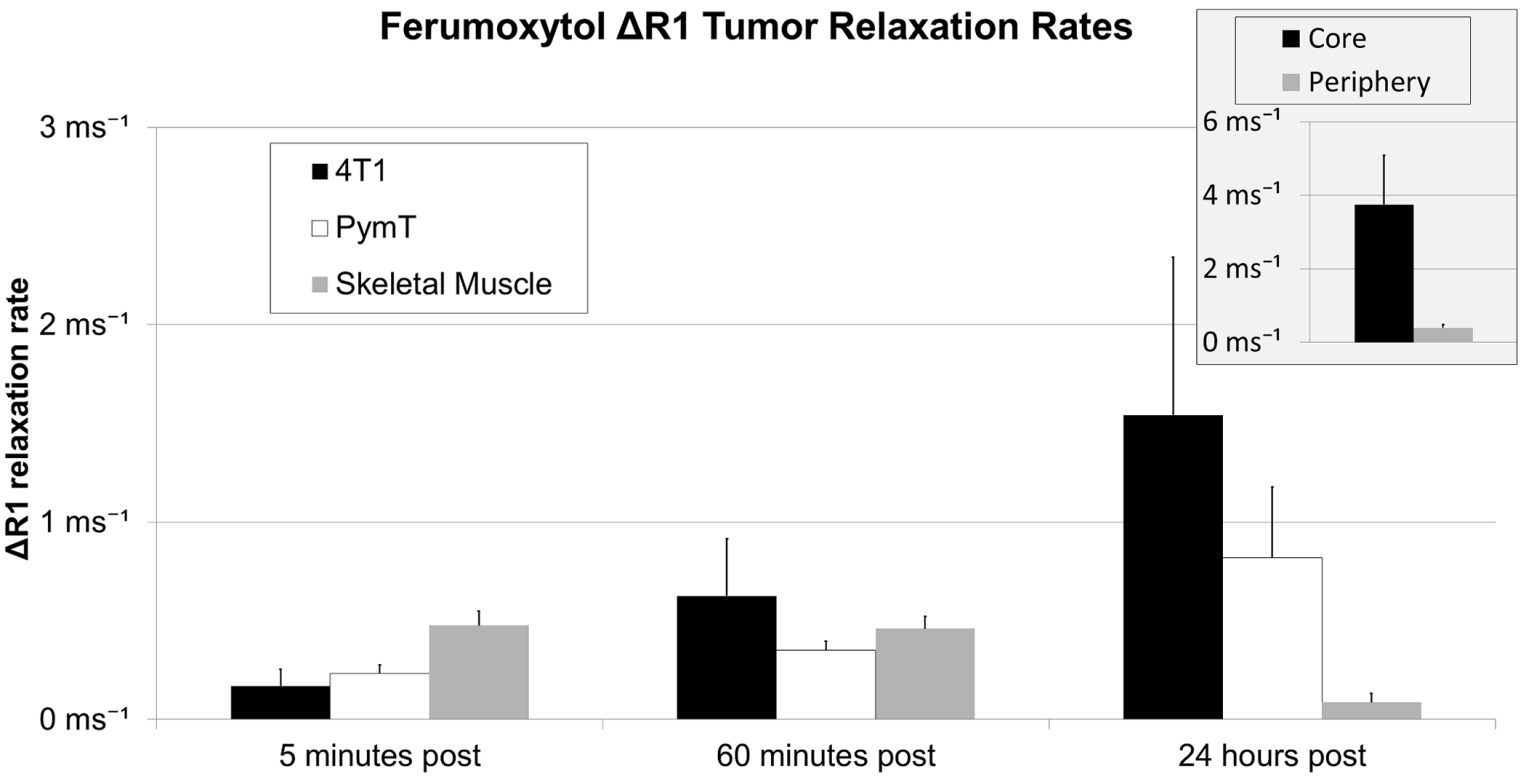
4T1 tumors with central necrosis exhibit significant T1-enhancement. Difference between pre- and post-contrast R1 relaxation rates (ΔR1 relaxation rates) of 4T1 tumors, PymT tumors as well as skeletal muscle. Data are displayed as mean data of six tumors in each group and standard deviations. Insert shows ΔR1 data of 4T1 tumor core versus periphery at 24 hours. Tumor ΔR1 data at 60 minutes and 24 hours post contrast are significantly different from baseline values (p<0.05).

Ferumoxytol injection caused significant long-lasting signal enhancement of iliac vessels in both MMTV-PymT and 4T1 animals on T1-weighted MR scans at 15 min and 24 hours p.i. (Fig 1). Both MMTV-PymT and 4T1 tumors demonstrated minimal T1 enhancement of the entire tumor at 1 hour p.i. without discernable differences between enhancement of the tumor core and center. However, at 24 h p.i. 4T1 tumors displayed a strongly hyperintense enhancement of the tumor center, while the tumor periphery showed only moderate enhancement. MMTV-PyMT tumors on the other hand, showed minor and homogenous T1-enhancement at both 1 and 24 hours p.i. Corresponding R1 rates of the tumor increased significantly compared to baseline with both 4T1 and MMTV-PyMT tumors (Fig 3). However, only 4T1 tumors demonstrated significantly increased R1 relaxation rates of the tumor core compared to the tumor periphery (4.38 ± 1.33 ms-1 and 0.63 ± 0.03 ms-1, respectively, p<0.001; Fig 3 insert). T2-weighted MR images showed marked T2-enhancement of 4T1 tumors and MMTV-PyMT tumors at 1 and 24 hours p.i. without discernable differences between tumor center and periphery.

The R1 relaxivity of ferumoxytol in 0.9% normal saline at 2 T and room temperature was 11.85 mM (Fe)^-1^s^-1^. Plasma ΔR1 was 125.8±10.1 s^-1^ 5 minutes p.i., 114.3±12.4 s^-1^ 60 minutes p.i. and 3.89±3.7 s^-1^ 24 hours p.i. Corresponding iron concentrations in plasma were 10.6±0.9 mM Fe at 5 minutes, 9.65±1.0 mM Fe at 60 minutes and 0.33±0.31 mM Fe at 24 hours. Assuming a mono-exponential loss of Fe in blood, we obtained a blood clearance rate of 0.15 h^-1^. As we could not faithfully measure blood in tumor secondary to the high ferumoxytol concentration in the blood, we used blood concentration curves to determine tumor leakage rates. This yielded a trans-endothelial leakage of 3.4±0.9x10^-3^ mL/min/100 cm^3^ for 4T1 tumors and 1.0±0.9x10^-3^ mL/min/100 cm^3^ for MMTV-PyMT tumors.

The average ΔR1 and ΔR2 in tumor and necrosis after 1 hour, 24 hour and 48 hour post injection are calculated and shown in Table 1.

**Table 1:** Average ΔR1 and ΔR2 in tumor and necrosis after 1 hour, 24 hour and 48 hour post injection.

	1 h	24 h (p.i.)	48 h (p.i.)
**ΔR1—tumor**	0.067	0.145	0.113
**ΔR1—necrosis**	0.058	0.373	0.328
**ΔR2—tumor**	258.2	509.2	518.8
**ΔR2—necrosis**	225.3	547.0	531.8

**Fig 4:**
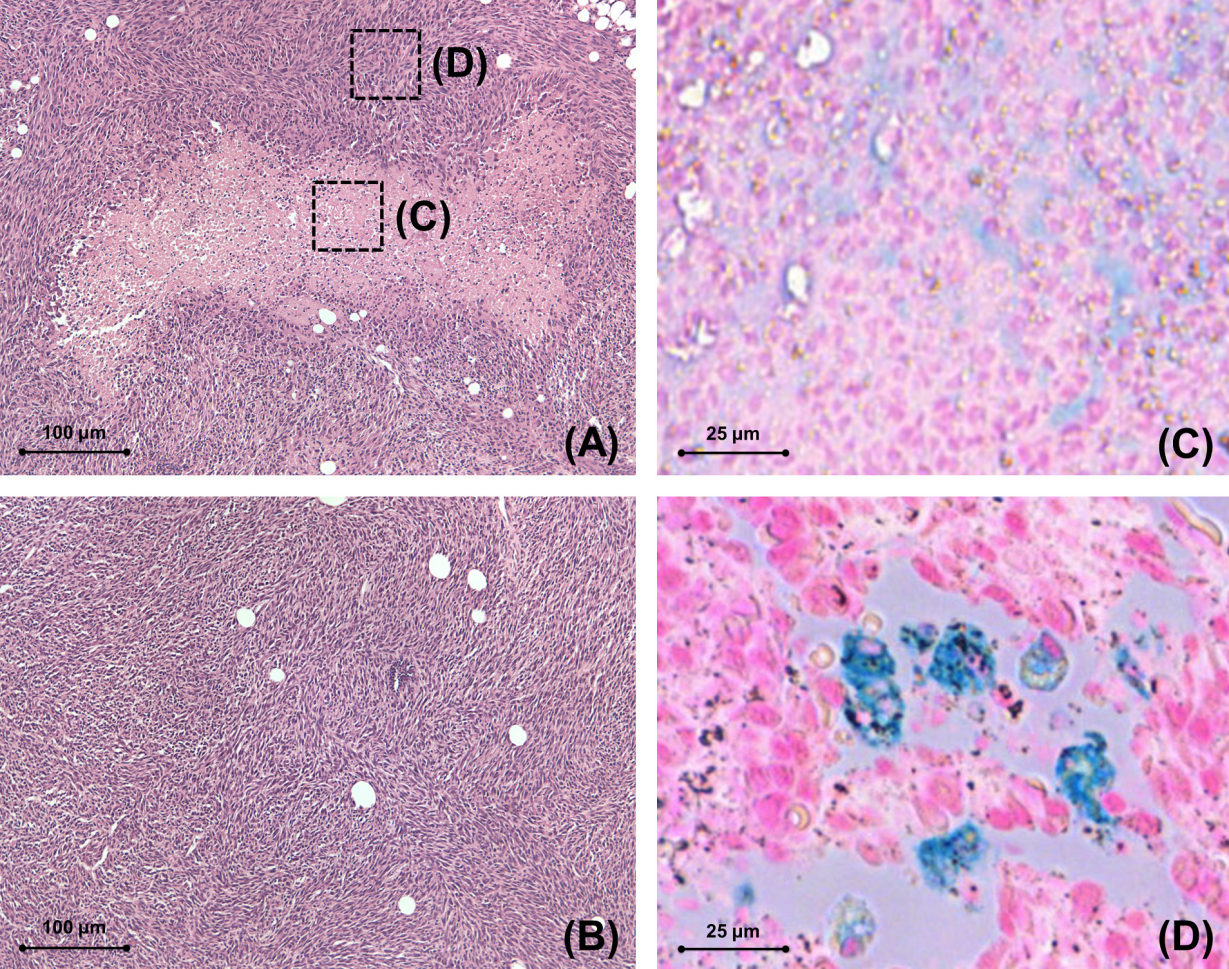
4T1 tumor T1-enhancement corresponds to clusters of free iron oxides in central necrosis. (A) H&E stain of 4T1 tumor shows a necrotic core (square C) and highly cellular tumor periphery (square D, 100x magnification). (B) H&E stain of PymT tumor shows uniformly heterogeneous tumor tissue without central necrosis (100x magnifications). (C) Prussian blue stain (400x magnification) of the necrotic core of the 4T1 tumor shows diffuse distribution blue (iron) staining consistent with iron oxide nanoparticles in a non-compartmentalized, extracellular localization. (D) Prussian blue stain (400x magnifications) of the peripheral area of the 4T1 tumor shows blue iron staining in cells.

Histological correlations showed a markedly heterogeneity in 4T1 tumor with a core comprised of cellular debris and leukocytes consistent with necrosis and a periphery comprised of tumor cells (Fig 4A). In the necrotic core, there was diffuse blue staining consistent with non-compartmentalized iron (Fig 4C). Prussian blue staining of tumor showed compartmentalized, cellular iron staining surrounding this interface between the core and the periphery (Fig 4D). MMTV-PymT tumors displayed a homogenous hyper cellular tumor (Fig 4B).

### Evaluation of tumor enhancement with monodisperse iron oxide nanoparticles

**Fig 5:**
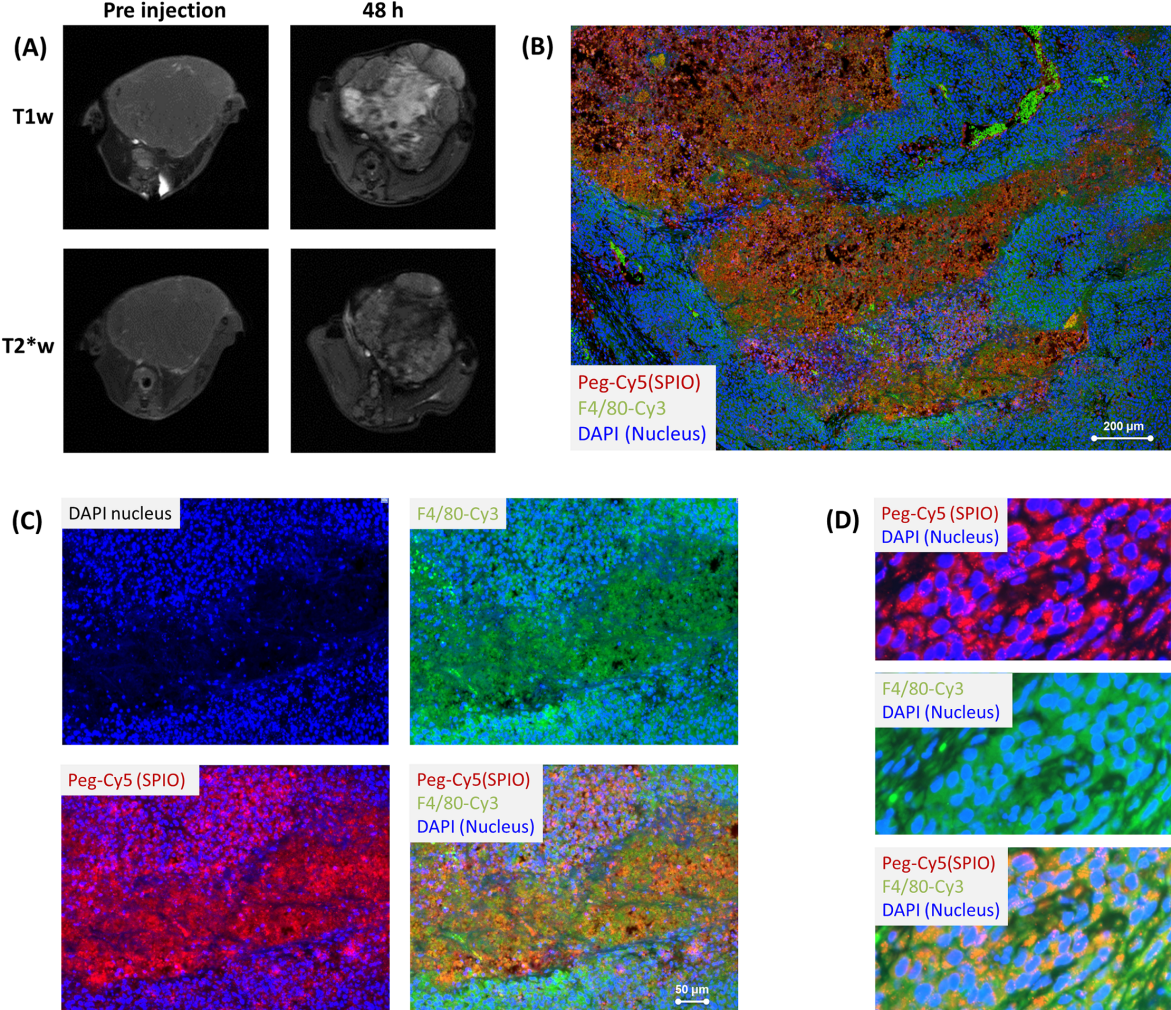
GEH121333 Tumor T1-enhancement corresponds to free iron oxides in central necrosis. (A) Axial T1- and T2*-weighted MR images of a representative PymT tumor before and 48 hours post injection (p.i.) of ferumoxytol at a dose of 0.5 mmol Fe/kg. Note homogenous tumors on pre-contrast images. The tumor center demonstrates hyperintense (bright) T1-enhancement and hypointense (dark) T2*-enhancement while the tumor periphery demonstrates hypointense enhancement on both sequences. (B) Corresponding histopathology (20x magnification) demonstrates areas of apparently free PEG-Cy5 (red) positive iron oxides in central tumor areas. F4/80 positive (green) macrophages outline these central areas. (C) Higher magnification (40x magnification) of the central tumor area confirms free PEG-Cy5 positive iron oxides (red) in central, necrotic tumor areas with low cell quantity (DAPI, blue). (D) Closer evaluation of peripheral tumor areas with high cell content demonstrate PEG-Cy5 (red) positive iron oxides co-localized to abundant F4/80 positive (green) macrophages.

In accordance with results described above, intravenous injection of the monodisperse iron oxide nanoparticle compound GEH121333 demonstrated significant positive (bright) T1-enhancement and significant negative (dark) T2-signal enhancement of central tumor areas (Fig 5), while presumably solid peripheral tumor portions demonstrated negative (dark) enhancement on both T1- and T2-weighted sequences.

Histological correlations showed an abundance of free (extracellular) iron oxide nanoparticles in central necrosis. The necrotic areas were outlined by a rim of macrophages, which contained iron oxides. More peripheral highly cellular tumor areas demonstrated iron oxide nanoparticles in macrophages and few interstitial iron oxides.

### Clinical translation

As a proof-of-concept for clinical translation of our observations, we investigated MR signal effects of ferumoxytol nanoparticles in four patients with malignant bone sarcomas. We used the iliac vessels as an internal standard for a proton-rich environment with extracellular USPIO and we used hematopoietic bone marrow as an internal standard for a highly cellular tissue with abundant macrophages and intracellular USPIO compartmentalization. The iliac vessels demonstrated significant T1-enhancement, while normal bone marrow demonstrated lack of T1-enhancement, consistent with our hypothesis that extracellular iron oxides in proton rich environment exhibit strong T1-signal effects while intracellularly compartmentalized iron oxides show diminished T1-signal effects. No difference between these differently compartmentalized iron oxides could be noted on T2-weighted MR scans.

**Fig 6:**
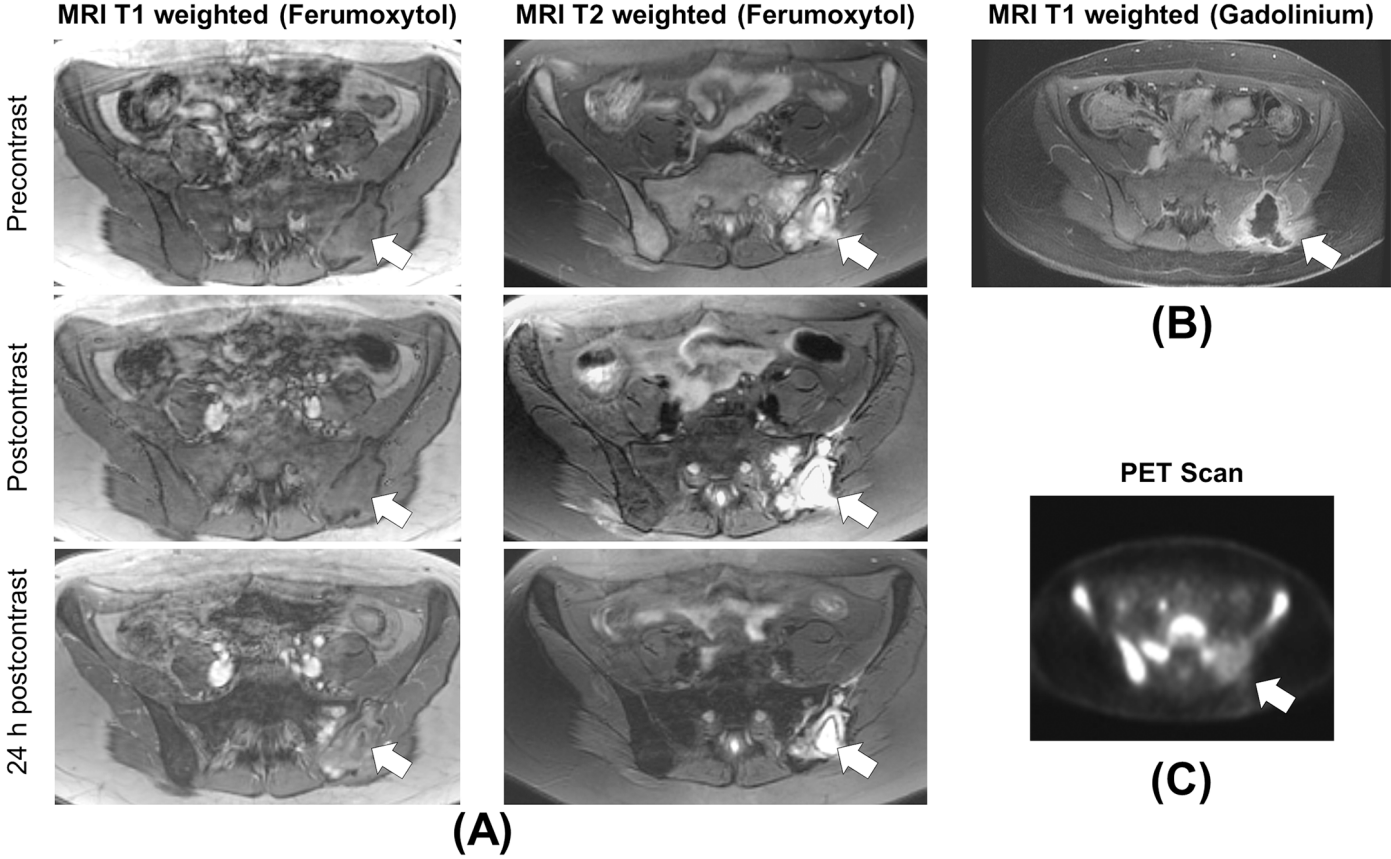
T1-enhancement of ferumoxytol in early necrosis of a Ewing’s sarcoma. A) T1- and T2 weighted MRI images of a 21 year old patient with metastatic Ewing’s sarcoma in the left sacrum and ilium (arrow). Normal bone marrow demonstrates marked T2-enhancement and minimal or no T1-enhancement at 24 h p.i., consistent with intracellular iron in bone marrow macrophages. Fluid-isointense liquefied necrosis demonstrates minimal peripheral rim enhancement. Blood in iliac vessels and T2-hyperintense tumor necrosis in the sacrum demonstrate marked T2-enhancement and marked T1-enhancement at 24 h p.i., consistent with extracellular iron. B) T1 weighed axial MRI image after intravenous injection of Gd-BOPTA demonstrates peripheral rim enhancement around the liquefied necrosis in the ilium, but interestingly no enhancement of the necrotic tumor area in the sacrum. C) PET image from the same area demonstrates highly cellular bone marrow after induction chemotherapy and relatively mild ^18^F-FDG uptake into the tumor (arrow).

**Fig 7:**
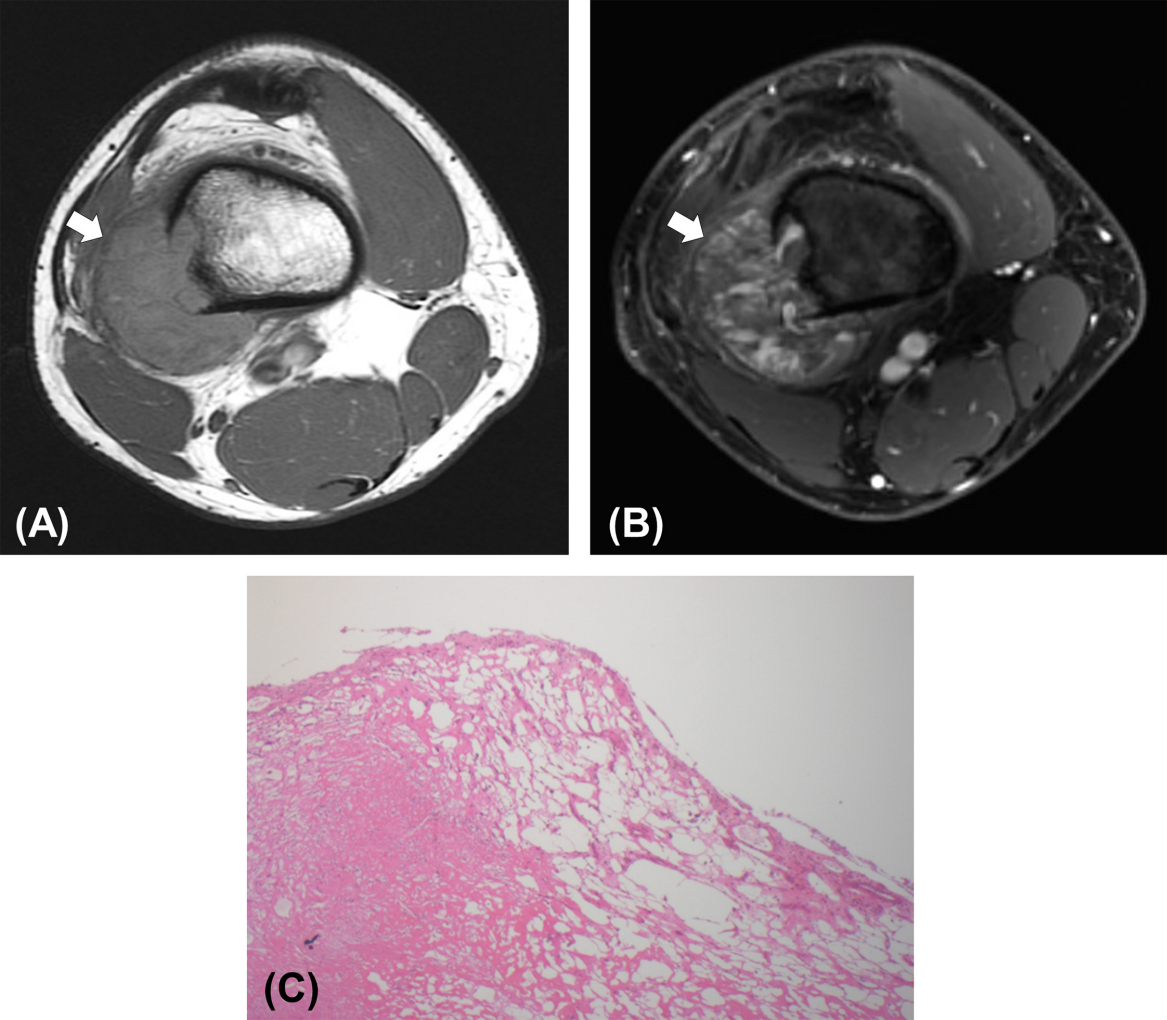
T1-enhancement of ferumoxytol in dilated vascular sinusoids of a teleangiectatic osteosarcoma. T1 weighted pre and post contrast images with corresponding histology. A) Non-contrast, nonfat-saturated, T1 weighted axial image of the femur shows a telangiectatic osteosarcoma (arrow). Tumor appears featureless. B) Fat-saturated, T1 weighted axial image of the same tumor after administration of 5 mg Fe/kg ferumoxytol. Tumor now shows hyperintense fluid-fluid levels in cystic-vascular areas. C) Corresponding histology shows findings compatible with telangiectatic osteosarcoma with numerous vascular sinusoids.

Accordingly, necrotic or cystic tumor areas with histopathological proven micro vessel supply, such as early tumor necrosis and telangiectatic tumor areas, demonstrated significant T1-enhancement. Solid tumor tissue demonstrated minimal or no T1-enhancement (Figs 6 and 7). On T2-weighted MR images, some areas of central necrosis did not show any iron oxide nanoparticle enhancement while other areas of necrotic/telangiectatic areas showed a negative (dark) T2-enhancement which did not show any difference in signal characteristics compared to solid tumor tissue.

## Discussion

Data showed that T1 weighted MR images provide important information about intracellular and extracellular compartmentalization of iron oxide nanoparticles in tumors. Ferumoxytol nanoparticles in tumor necrosis demonstrated marked T1 and T2-enhancement at 24 after injection, while intracellularly compartmentalized ferumoxytol nanoparticles demonstrated predominant T2, with little or no T1 enhancement. These *in vivo* findings are in accordance with previous *in vitro* studies on MR signal effects of intracellular and extracellular iron oxides [25].

Our data indicate that ferumoxytol-enhanced MRI may be more sensitive for the detection of an early necrosis compared to T2 weighted or Gd-DTPA enhanced T1 weighted MRI scans. The typical lack of contrast enhancement of tumor necrosis with Gd-DTPA is based on lack of tumor perfusion and the typical hyper-intensity on T2 weighted images is based on increased proton content, both of which may be only noted in advanced, liquefied necrosis [36]. Ferumoxytol tissue accumulation is largely dependent on microvascular permeability, which is increased in early necrosis. Hypoxia-induced increase in tumor microvascular permeability occurs earlier in the evolution of necrosis than decrease in blood volume or liquefaction [37]. This would have important clinical implications for estimating tumor aggressiveness, biopsy guidance, delivery of macromolecular therapeutic drugs and therapy monitoring. Tumor necrosis has been shown to be a valuable histological indicator of prognosis in many cancers including sarcomas [38] as well as malignancies of the colon, kidney and breast [39–41]. In a recent study in renal cancer, findings of tumor necrosis on conventional MRI were associated with disease progression and aggressive tumor pathology, irrespective of tumor size [42]. In sarcomas, biopsies after the initial diagnosis are used for therapy stratifications. An improved delineation of early necrosis could be used to direct biopsies to viable tumor areas, needed for tumor grading. Finally, a sensitive imaging technique for diagnosing early tumor necrosis could be used to accelerate imaging diagnosis of tumor chemotherapy response, spare patients from repeated ineffective therapies and assign non-responders to alternative treatment options.

Our data also showed in accordance with others that ferumoxytol causes little or no T1-enhancement in solid tumors. Turetschek *et al*. and Moore *et al*. postulated that USPIO-induced T1 and T2-enhancement in solid tumors on relatively early postcontrast scans is largely determined by the tumor’s blood volume, which is in the order of 5% for most cancers [23, 24, 26]. Our team added that a persistent T2-enhancement on delayed scans, 24h p.i. or later, without concomitant T1-effect is due to uptake and retention by tumor associated macrophages [1]. Normal hematopoietic bone marrow demonstrates a similar ferumoxytol enhancement pattern with significant T2-enhancement at 24 h p.i. and little or no T1-enhancement and may thus be used as an internal standard [30].

We acknowledge limitations of our study. In our animal model, we utilized a dose of ferumoxytol, which was fivefold higher than the FDA approved dose of iron oxide nanoparticles for patients (0.5 versus 0.1 mmol Fe/kg). Given the faster bio distribution and higher uptake efficiency for USPIO in rodent spleen and liver, these higher doses are recommended for rodent studies in order to provide similar contrast agent kinetics compared to humans [15, 27]. Our translational images proved to be comparable and thus using a lower dose for further human studies appears feasible. However, further studies in patients will need to evaluate the complex compartmentalization patterns of iron oxide nanoparticles in human tumors and their related MR signal effects.

## Conclusions

In summary, we have shown that extracellular iron oxides in early tumor necrosis lead to strong T1 and T2-enhancement while compartmentalized intracellular iron oxides in macrophages are characterized by predominant T2-enhancement with little or no T1-enhancement. This information is important for the interpretation of iron oxide enhanced MRI scans of malignant tumors, especially if conclusions are drawn between iron oxide MR enhancement and presence or quantities of macrophages in target tissues.

## References

[1] Daldrup-Link HE, Golovko D, Ruffell B, DeNardo DG, Castaneda R, Ansari C, et al MRI of tumor-associated macrophages with clinically applicable iron oxide nanoparticles. Clinical Cancer Research. 2011;17(17):5695–704. 10.1158/1078-0432.CCR-10-3420 21791632 PMC3166957

[2] Harisinghani MG, Barentsz J, Hahn PF, Deserno WM, Tabatabaei S, van de Kaa CH, et al Noninvasive detection of clinically occult lymph-node metastases in prostate cancer. New England Journal of Medicine. 2003;348(25):2491–9. 12815134 10.1056/NEJMoa022749

[3] Meier R, Henning TD, Boddington S, Tavri S, Arora S, Piontek G, et al Breast Cancers: MR Imaging of folate-receptor expression with the folate-specific nanoparticle P1133 1. Radiology. 2010. 10.1148/radiol.10090050 20413763

[4] Neuwelt EA, Várallyay CG, Manninger S, Solymosi D, Haluska M, Hunt MA, et al The potential of ferumoxytol nanoparticle magnetic resonance imaging, perfusion, and angiography in central nervous system malignancy: a pilot study. Neurosurgery. 2007;60(4):601–12. 17415196 10.1227/01.NEU.0000255350.71700.37

[5] Simon G, Link T, Wörtler K, Doebereiner F, Schulte-Frohlinde E, Daldrup-Link H, et al Detection of hepatocellular carcinoma: comparison of Gd-DTPA-and ferumoxides-enhanced MR imaging. European radiology. 2005;15(5):895–903. 15800773 10.1007/s00330-005-2669-1

[6] Corot C, Robert P, Idée J-M, Port M. Recent advances in iron oxide nanocrystal technology for medical imaging. Advanced drug delivery reviews. 2006;58(14):1471–504. 17116343 10.1016/j.addr.2006.09.013

[7] Shih YYI, Hsu YH, Duong TQ, Lin SS, Chow KPN, Chang C. Longitudinal study of tumor‐associated macrophages during tumor expansion using MRI. NMR in Biomedicine. 2011;24(10):1353–60. 10.1002/nbm.1698 22223366 PMC3733487

[8] Daldrup-Link HE, Simon GH, Brasch RC. Imaging of tumor angiogenesis: current approaches and future prospects. Current pharmaceutical design. 2006;12(21):2661–72. 16842165 10.2174/138161206777698774

[9] Pan D, Caruthers SD, Chen J, Winter PM, SenPan A, Schmieder AH, et al Nanomedicine strategies for molecular targets with MRI and optical imaging. Future medicinal chemistry. 2010;2(3):471–90. 10.4155/fmc.10.5 20485473 PMC2871711

[10] Santra S, Kaittanis C, Grimm J, Perez JM. Drug/Dye‐Loaded, Multifunctional Iron Oxide Nanoparticles for Combined Targeted Cancer Therapy and Dual Optical/Magnetic Resonance Imaging. small. 2009;5(16):1862–8. 10.1002/smll.200900389 19384879 PMC3057065

[11] Ahmed N, Fessi H, Elaissari A. Theranostic applications of nanoparticles in cancer. Drug Discovery Today. 2012;17(17):928–34. 22484464 10.1016/j.drudis.2012.03.010

[12] Ansari C, Tikhomirov GA, Hong SH, Falconer RA, Loadman PM, Gill JH, et al Development of Novel Tumor‐Targeted Theranostic Nanoparticles Activated by Membrane‐Type Matrix Metalloproteinases for Combined Cancer Magnetic Resonance Imaging and Therapy. Small. 2014;10(3):566–75. 10.1002/smll.201301456 24038954 PMC3946335

[13] Young JK, Figueroa ER, Drezek RA. Tunable nanostructures as photothermal theranostic agents. Annals of biomedical engineering. 2012;40(2):438–59. 10.1007/s10439-011-0472-5 22134466

[14] Li W, Salanitri J, Tutton S, Dunkle EE, Schneider JR, Caprini JA, et al Lower extremity deep venous thrombosis: evaluation with ferumoxytol-enhanced MR imaging and dual-contrast mechanism—preliminary experience. Radiology. 2007;242(3):873–81. 17325072 10.1148/radiol.2423052101

[15] Sigovan M, Boussel L, Sulaiman A, Sappey-Marinier D, Alsaid H, Desbleds-Mansard C, et al Rapid-Clearance Iron Nanoparticles for Inflammation Imaging of Atherosclerotic Plaque: Initial Experience in Animal Model 1. Radiology. 2009;252(2):401–9. 10.1148/radiol.2522081484 19703881

[16] Wang ZJ, Boddington S, Wendland M, Meier R, Corot C, Daldrup-Link H. MR imaging of ovarian tumors using folate-receptor-targeted contrast agents. Pediatric radiology. 2008;38(5):529–37. 10.1007/s00247-008-0764-6 18357444 PMC2745549

[17] Yilmaz A, Dengler MA, van der Kuip H, Yildiz H, Rösch S, Klumpp S, et al Imaging of myocardial infarction using ultrasmall superparamagnetic iron oxide nanoparticles: a human study using a multi-parametric cardiovascular magnetic resonance imaging approach. European heart journal. 2013;34(6):462–75. 10.1093/eurheartj/ehs366 23103659

[18] Triantafyllou M, Studer UE, Birkhäuser FD, Fleischmann A, Bains LJ, Petralia G, et al Ultrasmall superparamagnetic particles of iron oxide allow for the detection of metastases in normal sized pelvic lymph nodes of patients with bladder and/or prostate cancer. European Journal of Cancer. 2013;49(3):616–24. 10.1016/j.ejca.2012.09.034 23084842

[19] Daldrup-Link H, Coussens LM. MR imaging of tumor-associated macrophages. Oncoimmunology. 2012;1(4):507–9. 22754769 10.4161/onci.19456 PMC3382895

[20] Henning TD, Wendland MF, Golovko D, Sutton EJ, Sennino B, Malek F, et al Relaxation effects of ferucarbotran‐labeled mesenchymal stem cells at 1.5 T and 3T: Discrimination of viable from lysed cells. Magnetic Resonance in Medicine. 2009;62(2):325–32. 10.1002/mrm.22011 19353670 PMC2931823

[21] Smith BR, Kempen P, Bouley D, Xu A, Liu Z, Melosh N, et al Shape matters: intravital microscopy reveals surprising geometrical dependence for nanoparticles in tumor models of extravasation. Nano letters. 2012;12(7):3369–77. 10.1021/nl204175t 22650417 PMC3495189

[22] Daldrup-Link HE, Rydland J, Helbich TH, Bjørnerud A, Turetschek K, Kvistad KA, et al Quantification of Breast Tumor Microvascular Permeability with Feruglose-enhanced MR Imaging: Initial Phase II Multicenter Trial 1. Radiology. 2003;229(3):885–92. 14576446 10.1148/radiol.2293021045

[23] Turetschek K, Roberts TP, Floyd E, Preda A, Novikov V, Shames DM, et al Tumor microvascular characterization using ultrasmall superparamagnetic iron oxide particles (USPIO) in an experimental breast cancer model. Journal of magnetic resonance imaging. 2001;13(6):882–8. 11382948 10.1002/jmri.1126

[24] Moore A, Marecos E, Bogdanov A Jr, Weissleder R. Tumoral distribution of long-circulating dextran-coated iron oxide nanoparticles in a rodent model 1. Radiology. 2000;214(2):568–74. 10671613 10.1148/radiology.214.2.r00fe19568

[25] Simon GH, Bauer J, Saborovski O, Fu Y, Corot C, Wendland MF, et al T1 and T2 relaxivity of intracellular and extracellular USPIO at 1.5 T and 3T clinical MR scanning. European radiology. 2006;16(3):738–45. 16308692 10.1007/s00330-005-0031-2

[26] Turetschek K, Huber S, Floyd E, Helbich T, Roberts TP, Shames DM, et al MR Imaging Characterization of Microvessels in Experimental Breast Tumors by Using a Particulate Contrast Agent with Histopathologic Correlation 1. Radiology. 2001;218(2):562–9. 11161179 10.1148/radiology.218.2.r01fe37562

[27] Simon GH, von Vopelius-Feldt J, Fu Y, Schlegel J, Pinotek G, Wendland MF, et al Ultrasmall supraparamagnetic iron oxide-enhanced magnetic resonance imaging of antigen-induced arthritis: a comparative study between SHU 555 C, ferumoxtran-10, and ferumoxytol. Invest Radiol. 2006;41(1):45–51. 16355039 10.1097/01.rli.0000191367.61306.83

[28] Lu M, Cohen MH, Rieves D, Pazdur R. FDA report: Ferumoxytol for intravenous iron therapy in adult patients with chronic kidney disease. Am J Hematol. 2010;85(5):315–9. 10.1002/ajh.21656 20201089

[29] McLachlan SJ, Morris MR, Lucas MA, Fisco RA, Eakins MN, Fowler DR, et al Phase I clinical evaluation of a new iron oxide MR contrast agent. Journal of Magnetic Resonance Imaging. 1994;4(3):301–7. 8061425 10.1002/jmri.1880040313

[30] Shi Q, Pisani LJ, Lee YK, Messing S, Ansari C, Bhaumik S, et al Evaluation of the novel USPIO GEH121333 for MR imaging of cancer immune responses. Contrast media & molecular imaging. 2013;8(3):281–8. 10.1002/cmmi.1526 23606432 PMC3662997

[31] Daldrup H, Shames DM, Wendland M, Okuhata Y, Link TM, Rosenau W, et al Correlation of dynamic contrast-enhanced MR imaging with histologic tumor grade: comparison of macromolecular and small-molecular contrast media. AJR American journal of roentgenology. 1998;171(4):941–9. 9762973 10.2214/ajr.171.4.9762973

[32] Guy C, Cardiff R, Muller W. Induction of mammary tumors by expression of polyomavirus middle T oncogene: a transgenic mouse model for metastatic disease. Molecular and cellular biology. 1992;12(3):954–61. 1312220 10.1128/mcb.12.3.954-961.1992 PMC369527

[33] Varticovski L, Hollingshead MG, Robles AI, Wu X, Cherry J, Munroe DJ, et al Accelerated preclinical testing using transplanted tumors from genetically engineered mouse breast cancer models. Clinical cancer research. 2007;13(7):2168–77. 17404101 10.1158/1078-0432.CCR-06-0918

[34] Roberts TP. Physiologic measurements by contrast‐enhanced MR imaging: Expectations and limitations. Journal of Magnetic Resonance Imaging. 1997;7(1):82–90. 9039597 10.1002/jmri.1880070112

[35] Klenk C, Gawande R, Uslu L, Khurana A, Qiu D, Quon A, et al Ionising radiation-free whole-body MRI versus< sup> 18 F-fluorodeoxyglucose PET/CT scans for children and young adults with cancer: a prospective, non-randomised, single-centre study. The lancet oncology. 2014;15(3):275–85. 10.1016/S1470-2045(14)70021-X 24559803

[36] Lim HS, Jeong YY, Kang HK, Kim JK, Park JG. Imaging features of hepatocellular carcinoma after transcatheter arterial chemoembolization and radiofrequency ablation. American Journal of Roentgenology. 2006;187(4):W341–W9. 16985104 10.2214/AJR.04.1932

[37] Fukumura D, Duda DG, Munn LL, Jain RK. Tumor Microvasculature and Microenvironment: Novel Insights Through Intravital Imaging in Pre‐Clinical Models. Microcirculation. 2010;17(3):206–25. 10.1111/j.1549-8719.2010.00029.x 20374484 PMC2859831

[38] Neuville A, Chibon F, Coindre J-M. Grading of soft tissue sarcomas: from histological to molecular assessment. Pathology-Journal of the RCPA. 2014;46(2):113–20. 10.1097/PAT.0000000000000048 24378389

[39] Pollheimer MJ, Kornprat P, Lindtner RA, Harbaum L, Schlemmer A, Rehak P, et al Tumor necrosis is a new promising prognostic factor in colorectal cancer. Human pathology. 2010;41(12):1749–57. 10.1016/j.humpath.2010.04.018 20869096

[40] Pichler M, Hutterer GC, Chromecki TF, Jesche J, Kampel-Kettner K, Rehak P, et al Histologic tumor necrosis is an independent prognostic indicator for clear cell and papillary renal cell carcinoma. American journal of clinical pathology. 2012;137(2):283–9. 10.1309/AJCPLBK9L9KDYQZP 22261455

[41] Richards CH, Mohammed Z, Qayyum T, Horgan PG, McMillan DC. The prognostic value of histological tumor necrosis in solid organ malignant disease: a systematic review. Future Oncology. 2011;7(10):1223–35. 10.2217/fon.11.99 21992733

[42] Beddy P, Genega EM, Ngo L, Hindman N, Wei J, Bullock A, et al Tumor necrosis on magnetic resonance imaging correlates with aggressive histology and disease progression in clear cell renal cell carcinoma. Clinical genitourinary cancer. 2014;12(1):55–62. 10.1016/j.clgc.2013.07.006 24145001 PMC4364293

